# Medicinal Herbs and Their Active Compounds for Fatty Liver Diseases

**DOI:** 10.1155/2017/3612478

**Published:** 2017-12-07

**Authors:** Chang Gue Son, Zhang Wei, H. Balaji Raghavendran, Jing-Hua Wang, Elzbieta Janda

**Affiliations:** ^1^Liver & Immunology Research Center, Daejeon Oriental Hospital of Daejeon University, Daejeon, Republic of Korea; ^2^Shanghai University of Traditional Chinese Medicine, Shanghai, China; ^3^Department of Orthopedic Surgery, University of Malaya, Faculty of Medicine, Kuala Lumpur, Malaysia; ^4^Key Laboratory of Xin'an Medicine, Ministry of Education, Clinical College of TCM, Anhui University of TCM, Hefei, Anhui Province, China; ^5^Department of Health Sciences, Magna Graecia University, Campus Germaneto, Catanzaro, Italy

Fatty liver disease (FLD), also called commonly as fatty liver or hepatic steatosis, is a condition of the excessive accumulation of lipids in hepatocytes. The prevalence of FLD in the general population ranges from 10% to 24% in various countries [1]. Fatty liver is generally classified as alcoholic steatosis (alcoholic FLD) or nonalcoholic fatty liver disease (NAFLD), depending on the contribution of alcohol to the FLD pathogenesis [2]. Due to decreased number of hepatic viruses carriers and increased population with obesity, FLD has become the most common cause of abnormal liver function tests in developed countries recently [3].

Fatty liver is a leading step to chronic liver diseases including steatohepatitis (nonalcoholic steatohepatitis, NASH, and alcoholic steatohepatitis, ASH), liver fibrosis, and cirrhosis worldwide [4]. In addition, FLD is also associated with other diseases such as metabolic syndrome and diabetes mellitus [5]. FLD has a complex pathology involving the imbalance between lipogenesis and lipolysis, followed by inflammatory response [6]. The high prevalence of FLD is considered as a medical issue worldwide; yet there are no currently available drug-based therapies. For this reason medicinal herbs-derived remedies are emerging as potential therapeutics against FLD due to high efficacy and low risk of side effects [7, 8].

This special issue is an attempt to contribute to the knowledge on CAM treatments for fatty liver diseases and its associated disorders. We called for articles that have explored effectiveness and mechanisms of medicinal herbs and their compounds on FLD. A collection of seven original research articles are presented, which address the animal (six articles) or cell-based (one article) pharmacological effects of herbal drugs or their compounds on FLD. This issue presets herbal resources which consisted of three multiherbal formulae (Dahuang Zexie Decoction, Yinchen Linggui Zhugan Decoction, and Seyoeum), three of medicinal plants* (Artemisia iwayomogi *plus* Curcuma longa, *Salvia-Nelumbinis Naturalis, and* Euphorbia kansui),* and one citrus bioflavonoid (Hesperidin) against NAFLD, nonalcoholic steatohepatitis (NASH), and lipid metabolic disorders, respectively.

This special issue provides valuable information to practitioners and researchers working in the field of FLD regarding new potential medicinal herbs and pathophysiological features of multitargets in FLD. We hope that it will become their inspiration or reference to develop new therapeutic strategies against FLD based on herbal-derived multidrugs and bioactive compounds.
